# Combined Body Mass Index and Waist-to-Height Ratio and Its Association with Lifestyle and Health Factors among Spanish Children: The PASOS Study

**DOI:** 10.3390/nu14020234

**Published:** 2022-01-06

**Authors:** Maria del Mar Bibiloni, Laura Gallardo-Alfaro, Santiago F. Gómez, Julia Wärnberg, Maddi Osés-Recalde, Marcela González-Gross, Narcís Gusi, Susana Aznar, Elena Marín-Cascales, Miguel González-Valeiro, Lluís Serra-Majem, Nicolás Terrados, Marta Segu, Camille Lassale, Clara Homs, Juan Carlos Benavente-Marín, Idoia Labayen, Augusto G. Zapico, Jesús Sánchez-Gómez, Fabio Jiménez-Zazo, Pedro E. Alcaraz, Marta Sevilla-Sánchez, Estefanía Herrera-Ramos, Susana Pulgar, Clara Sistac, Helmut Schröder, Cristina Bouzas, Josep A. Tur

**Affiliations:** 1 Research Group on Community Nutrition and Oxidative Stress, University of Balearic Islands-IUNICS & IDISBA, 07122 Palma de Mallorca, Spain; mar.bibiloni@uib.es (M.d.M.B.); lauragala3@gmail.com (L.G.-A.); cristina.bouzas@uib.es (C.B.); 2 Centro de Investigación Biomédica en Red Fisiopatología de la Obesidad y la Nutrición (CIBEROBN), Institute of Health Carlos III, 28029 Madrid, Spain; jwarnberg@uma.es (J.W.); marcela.gonzalez.gross@upm.es (M.G.-G.); lluis.serra@ulpgc.es (L.S.-M.); 3 Gasol Foundation, Sant Boi de Llobregat, 08830 Barcelona, Spain; sgomez@gasolfoundation.org (S.F.G.); choms@gasolfoundation.org (C.H.); 4 GREpS, Health Education Research Group, Nursing and Physiotherapy Department, University of Lleida, 25008 Lleida, Spain; 5 Epi-Phaan Research Group, Institute of Biomedical Research of Malaga (IBIMA), Universidad de Málaga, 29016 Malaga, Spain; jc.benaventemarin@gmail.com; 6 ELIKOS Group, Institute for Innovation and Sustainable Development in Food Chain (IS-FOOD), Department of Health Sciences, Public University of Navarre, 31008 Pamplona, Spain; maddiosre@gmail.com (M.O.-R.); idoia.labayen@unavarra.es (I.L.); 7 ImFINE Research Group, Department of Health and Human Performance, Universidad Politécnica de Madrid, 28040 Madrid, Spain; agarciaz@ucm.es; 8 Physical Activity and Quality of Life Research Group (AFYCAV), Faculty of Sport Sciences, University of Extremadura, 10003 Caceres, Spain; ngusi@unex.es (N.G.); jesanchezg@unex.es (J.S.-G.); 9 PAFS Research Group, Faculty of Sports Sciences, La Mancha-Toledo Campus, University of Castilla, 45071 Toledo, Spain; susana.aznar@uclm.es (S.A.); fabio_avila@hotmail.es (F.J.-Z.); 10 Research Center for High Performance Sport, San Antonio Catholic University of Murcia, 30107 Murcia, Spain; emarin@ucam.edu (E.M.-C.); palcaraz@ucam.edu (P.E.A.); 11 Faculty of Sport Sciences, San Antonio Catholic University of Murcia, 30107 Murcia, Spain; 12 Faculty of Sports Sciences and Physical Education, Universidade da Coruña, 15008 A Coruña, Spain; miguel.gonzalez.valeiro@udc.es (M.G.-V.); marta.sevilla@udc.es (M.S.-S.); 13 Research Institute of Biomedical and Health Sciences (IUIBS), University of Las Palmas de Gran Canaria, 35016 Las Palmas, Spain; estefania.herrera@ulpgc.es; 14 Preventive Medicine Service, Centro Hospitalario Universitario Insular Materno Infantil (CHUIMI), Canarian Health Service, 35016 Las Palmas, Spain; 15 Regional Unit of Sports Medicine of Principado de Asturias, Municipal Sports Foundation of Aviles, 33401 Aviles, Spain; nterrados@aviles.es (N.T.); susanapulgarmunoz@gmail.com (S.P.); 16 Probitas Foundation, 08022 Barcelona, Spain; marta.segu@fundacionprobitas.org (M.S.); clara.sistac@fundacionprobitas.org (C.S.); 17 CIBER de Epidemiología y Salud Pública (CIBERESP), Instituto de Salud Carlos III, 28049 Madrid, Spain; classale@imim.es (C.L.); hschroeder@imim.es (H.S.); 18 Cardiovascular Risk and Nutrition Research Group, Hospital del Mar Institute for Medical Research, 08003 Barcelona, Spain; 19 Global Research on Wellbeing (GRoW), Blanquerna Faculty of Health Sciences, Ramon Llull University, 08022 Barcelona, Spain; 20 Department of Didactics of Language, Arts and Physical Education, Universidad Complutense de Madrid, 28040 Madrid, Spain

**Keywords:** Mediterranean diet, lifestyle, children, adolescents, PASOS

## Abstract

Background and Aims: The World Health Organization recommended simultaneous measurement of body mass index (BMI) and waist circumference (WC) and suggested joint use to predict disease risks. The aim of this study was to assess the prevalence of BMI and waist-to-height ratio (WHtR) categories among Spanish children and adolescents, as well as their associations with several lifestyle factors. Methods: Cross-sectional analysis of 8–16-year-old children and adolescents (*n* = 3772) were included in the PASOS nationwide representative study. Children/adolescents and their mothers/female caregivers answered a questionnaire on lifestyle and health factors. Child/adolescent anthropometrics were measured. Four combined BMI-WHtR disease risk categories were built. Results: A third of participants showed combined BMI-WHtR categories with high disease risk (12.3% ‘increased risk’, 9.7% ‘high risk’, 14.3% ‘very high risk’). Participants in the ‘very high risk’ group were less likely to be females (odds ratio 0.63; 95% CI: 0.52–0.76) and adolescents (0.60; 95% CI: 0.49–0.72), to practice ≥60 min/day of moderate-vigorous physical activity (MVPA) (0.73; 95% CI: 0.57–0.93), and to watch <120 min/day of total screen time on weekdays (0.61; 95% CI: 0.49–0.76). Mothers of participants in the ‘very high risk’ group were less likely to have a high educational level, be in the overweight or normal range, have never smoked or were former smokers, and watch <120 min/day of total screen time on weekends. Participants in the ‘increased’ and ‘high risk’ categories had mothers with normal weight and ≥60 min/day of MVPA. Participants in the ’high risk’ group did not achieve ≥60 min/day of MVPA and showed lower adherence to the Mediterranean diet. Conclusions: Adherence to a healthy lifestyle in children and adolescents, but also in their mothers/female caregivers during offspring’s childhood and adolescence, is associated with low BMI-WHtR disease risk.

## 1. Introduction

Childhood and adolescent obesity is a major public health problem, and its prevalence has increased substantially in the last decades in both developed and developing countries [1]. In all age ranges, obesity is associated with several adverse health effects (e.g., dyslipidemia, insulin resistance, type 2 diabetes, and hypertension, as well as social and psychological problems) [2,3].

Body mass index (BMI) is the most used anthropometric predictor of overweight and obesity in research and clinical practice. However, BMI cannot distinguish between fat mass and fat-free mass and high BMI does not necessarily reflect increased adiposity [4]. Waist circumference (WC) and waist-to-height ratio (WHtR) provide relevant information on fat distribution and show the degree of abdominal obesity [5,6]. Evidence revealed that abdominal obesity is a better predictor of the presence of cardiometabolic risk factors than obesity evaluated by means of BMI [7]. A substantial proportion of children and adolescents with normal BMI had abdominal obesity [8,9,10,11], and adverse metabolic profiles appear to be more prevalent in children and adolescents with abdominal obesity than in those with overweight and obesity [7].

The World Health Organization (WHO) recommended simultaneous measurement of BMI and WC and suggested their joint use to predict disease risks [12]. However, WC does not reflect differences in height and thus may under- or over-evaluate the risks for short and tall individuals, respectively [13]. In contrast, WHtR does not require population-specific reference values or sex- or age-specific reference criteria, and it can perfectly track changes across ages in children and adolescents [14]. WHtR has a more profiled relation with several lifestyle factors as well as with psychological health in adults than BMI [15]. Although many studies have examined the association between health behaviors and BMI in children and adolescents, studies on health behaviors and combined BMI-WHtR are scarce [8,9,10,11]. Therefore, it is important to obtain more knowledge about factors related to combining BMI-WHtR categories.

The aim of this study was to assess the prevalence of BMI-WHtR categories among Spanish children and adolescents, as well as to assess their associations with several lifestyle factors.

## 2. Methods

### 2.1. Study Design

Cross-sectional analysis within the frame of the PASOS study (Physical Activity, Sedentarism and Obesity in Spanish Youth), a national representative, observational and multicenter research, was conducted. Details of the PASOS study protocol have been fully described [16].

### 2.2. Participants, Recruitment, and Ethics

Participants of the PASOS study were 8–16-year-old children and adolescents enrolled in a participating school. Students with an intellectual disability that prevented a response to the lifestyle questionnaires were excluded. Each case was evaluated with the corresponding teachers and parents or caregivers before exclusion.

Participants were recruited from March 2019 to February 2020 in 242 primary and secondary schools in 121 localities from each of the 17 Spanish regions (Ceuta and Melilla, two autonomous cities in North Africa with less than 0.8% of the total Spanish population aged 8–16 years were not included for logistical reasons). Lifestyle data of children/adolescents were self-reported online at participating schools. Parental sociodemographics, lifestyle data, and health habits were also obtained. Recruiters were trained to minimize the inter-observer coefficients of variation.

All parents and legal tutors of participants provided written informed consent. The study protocol and procedures were approved by the Ethics Committee of the Fundació Sant Joan de Déu, Barcelona, Spain. The trial was registered in 2019 at the International Standard Randomized Controlled Trial (ISRCT; https://doi.org/10.1186/ISRCTN34251612 (accessed on 19 July 2021) with the number 34251612.

### 2.3. Anthropometric Variables

Anthropometric variables for each child/adolescent were measured by previously trained field researchers with a background in physical education, nutrition, or other health sciences following the WHO standardized protocol [17], and after they completed a 1-day training session on the project methodology, hosted by the Gasol Foundation [16]. Reliability was previously checked by means of a validation pilot study [16]. Body weight and height were measured with the participant in light clothing, without shoes. Waist circumference was measured standing by placing a tape measure around the middle, just above the hipbones, making sure the tape was horizontal around the waist, keeping the tape snug around the waist but not compressing the skin, and measuring the waist just after breathing out. The measurements were performed using an electronic SECA 899 scale (recorded to the nearest 100 g), a portable SECA 217 stadiometer (to the nearest 1 mm), and a flexible non-stretch SECA 201 metric tape (to the nearest 1 mm), respectively. The BMI (kg/m^2^) was calculated using weight and height measures. The BMI Z-score of each child/adolescent was calculated according to the WHO 2007 growth standards and the reference for children and adolescents aged 5 to 19 years [18], and the weight status category of each child/adolescent was determined by age and sex according to the following BMI Z-score cutoffs: severe obesity > 3 standard deviation (SD); obesity (OB) > 2SD and ≤3SD; overweight (OW) > 1SD and ≤2SD; normal-weight (NW) ≥ −2SD and ≤1SD; underweight < −2SD and ≥−3SD; and severe underweight < −3SD. For this study, children/adolescents were categorized into three groups based on their BMI category: OB, BMI Z-score > 3 (labelled BMI_OB_); OW, 2 > BMI Z-score ≤ 3 (labelled BMI_OW_); NW, BMI Z-score ≤ 2 (labelled BMI_NW_). The WHtR was calculated as WC/height, with both measurements expressed in cm. Children/adolescents were categorized into two groups using the cut-off of 0.5: WHtR < 0.5, abdominal non-obesity (labeled WHtR_NO_), and WHtR ≥ 0.5, abdominal obesity (labeled WHtR_OB_) [19]. The two categories obesity (WHtR_NO_ and WHtR_O_) were collapsed to the ‘very high risk’ category due to small numbers (*n* = 51).

### 2.4. BMI-WHtR Classification

Children/adolescents were categorized into four groups based on their BMI and WHtR category and according to the risk for type 2 diabetes, hypertension, and cardiovascular diseases associated with OW and OB: ‘low risk’, BMI_NW_-WHtR_NO_; ‘increased risk’, BMI_OW_-WHtR_NO_; ‘high risk’, BMI_NW-OW_-WHtR_OB_; and ‘very high risk’, BMI_OB_-WHtR_NO-OB_ [20].

### 2.5. Assessment of Mediterranean Diet Adherence

The 16-item KIDMED questionnaire (dichotomous response options: Yes/No), created to estimate adherence to the Mediterranean diet (MedDiet) in children and young adults, based on the principles that sustain Mediterranean dietary patterns and those that undermine it, was administered [21]. An affirmative answer to items denoting lower adherence was assigned a value of −1 (4 items) and those related to higher adherence were scored +1 (12 items). This index was then used to classify subjects into three categories according to their adherence to the MedDiet: “low” = −4 to 3 points; “moderate” = 4 to 7 points; and “optimal” = 8 to 12 points.

### 2.6. Physical Activity and Sedentary Behavior

The PAU-7S, a 7-item self-reported questionnaire, was used to assess physical activity levels in each participating child/adolescent as described in the study protocol [16]. Only the average daily time (expressed in minutes) spent in moderate-vigorous physical activity (MVPA) was determined. Children/adolescents were categorized into two groups (<60 min/day; ≥60 min/day) based on their compliance with MVPA daily recommendations [22].

Sedentary behavior was calculated using the Screen-time Sedentary Behaviour Questionnaire [23], which asks about time spent in four activities: (1) watching TV, (2) playing computer games, (3) playing console (video) games, and (4) using a mobile phone, separately for weekdays and weekends. Children/adolescents were categorized into two groups (<120 min/day; ≥120 min/day) based on their compliance with screen time recommendations proposed by the American Academy of Pediatrics [24].

### 2.7. Obesogenic Score

The cumulative effect of obesogenic behaviors was assessed by means of a modified version of the obesogenic behavior score previously described [25]. The score was calculated by giving +1 point for low physical activity (<60 min/day), +1 point for high screen time (≥120 min/day), and +1 point for skipping daily breakfast. The final score ranged from 0 to 3 points, with higher scores indicating cumulative unhealthy obesogenic behaviors.

### 2.8. Mothers/Female Legal Guardians’ Outcomes

Two sets of questionnaires were delivered to each participating child/adolescent, to be answered separately by up to two parents/caregivers. However, in the present study, only the information obtained by mothers/females’ legal guardians was used.

The validated REGICOR (REgistre GIroní del COR) Short Physical Activity Questionnaire [26] and the following standardized questions were included: weight (kg), height (cm), smoking habit (smoker; former smoker; never smoker), and educational level (primary/illiterate; secondary education; university education). The mothers/female caregivers were classified according to their BMI into three groups: NW, BMI < 25 kg/m^2^; OW, BMI ≥ 25 to <30 kg/m^2^; and OB, BMI ≥ 30 kg/m^2^. Mothers/female caregivers were classified according to their total physical activity practice into two groups using the cut-off of 300 METs·min/week (<300 METs·min/week; ≥300 METs·min/week) [27,28]. Sedentary behavior was determined as total hours per day in front of a screen, and mothers/female caregivers were classified into two groups using the cut-off of <120 min/day (<120 min/day; ≥120 min/day). Dietary habits of mothers/female caregivers were assessed by the Short Diet Quality Screener (SDQS) questionnaire based on the frequency of consumption of 18 foods/food groups [29]. For each mother/female legal guardian, SDQS terciles were calculated.

### 2.9. Statistics

The present analysis included 3772 participants with full information of the main interesting outcomes (i.e., weight, height, and WC). Information from participants with two mothers/female caregivers (second couples) were excluded to avoid duplication of the same child/adolescent within the same category, which may introduce potential bias in the statistical analysis.

Analyses were performed with the Statistical Package for the Social Sciences version 25.0 (IBM SPSS Statistics for Windows, Chicago, IL, USA). Categorical variables were presented as frequencies and continuous variables as mean and SD. Significant differences in categorical variables were tested by the Chi-squared test, whereas in continuous variables among the four groups by analysis of variance test (ANOVA). Equality of variances was assessed with Levene’s test. Bonferroni was used as a post-hoc analysis. Logistic regression analysis with the estimation of the corresponding odds ratio (OR) and the 95% confidence interval (CI) were calculated to: (1) examine the association between each BMI-WHtR category (dependent variable), with ‘low risk’ (BMI_NW_-WHtR_NO_) category as reference, and lifestyle characteristics (independent variables); (2) examine the association between children’s “yes” answers in the KIDMED questionnaire (dependent variable) with a “no” answer as the reference variable, and each BMI-WHtR category (independent variable). Logistic regression analyses were adjusted for sex and age, unless the variable was the one of interest, to control for potential confounders. Results were considered statistically significant if *p*-value (2 tailed) <0.05.

## 3. Results

Applying the WHO 2007 BMI criteria, the prevalence of NW, OW, and OB were 65.0%, 20.7%, and 14.3%, respectively. The prevalence of abdominal obesity defined as WHtR ≥0.5 was 22.7% (2.1% of NW, 40.4% of OW, and 90.6% of OB participants) (data not shown). More than a third of the participants showed combined BMI-WHtR categories with high disease risk: 12.3% at ‘increased risk’, 9.7% at ‘high risk’, and 14.3% at ‘very high risk’. Only 51 participants had general obesity but not abdominal obesity. Table 1 shows the sample characteristics by BMI-WHtR categories. There were significant differences in the following variables across BMI-WHtR groups: school grade, sex, the achievement of the recommended MVPA, the achievement of the recommended screen time on weekdays, KIDMED index, and obesogenic behavior score. Table 2 shows maternal characteristics by participants’ BMI-WHtR categories. There were significant differences in the following mothers’ variables across BMI-WHtR groups: education level, BMI categories, SDQS terciles, smoking habit, the achievement of the recommended screen time in mothers on weekdays, and the achievement of the recommended physical activity on weekdays. There were also differences between the percentages of the studied population developing PA >300 METs × min/day.

The binary logistic regression analysis adjusted by age and sex to determine the associated lifestyle and health factors with child/adolescent BMI-WHtR ‘increased’, ‘high’, and ‘very high risk’ categories are shown in Table 3. BMI-WHtR ‘increased’ and ‘very high risk’ groups were negatively associated with adolescence (≥12 years). Individuals in the ‘very high risk’ group were less likely to be females. The BMI-WHtR ‘high risk’ group was less likely to achieve the recommendation of at least 60 min/day of MVPA but also to be less likely to have a moderate and optimal adherence to the MedDiet. Participants with a BMI-WHtR ‘very high risk’ were also less likely to achieve the recommendation of at least 60 min/day of MVPA, and the recommendation of <120 min/day of total screen time. Finally, BMI-WHtR ‘high’ and ‘very high risk’ categories were positively associated with the obesogenic behavior score.

Children/adolescents with elevated BMI-WHtR disease risk were less likely to have mothers with a weight in the normal range (<25 kg/m^2^). Participants in the ‘increased’ and ‘very high risk’ category were also less likely to have mothers with OW. Participants in the ‘very high risk’ group were also less likely to have mothers with a higher education level, mothers who never smoked or were former smokers, mothers with a high SDQS score, and mothers who achieved the recommendation of <120 min/day of total screen time on weekdays. Participants in the ‘increased’ and ‘high risk’ categories were less likely to have mothers who achieved the recommendation of at least 60 min/day of MVPA; no significant differences were found in the ‘very high risk’ category.

Table 4 shows the results of the logistic regression for “yes” answers in the KIDMED questionnaire. Skipping breakfast was positively associated with ‘increased’, ‘high’, and ‘very high risk’ groups, and consumption of dairy products for breakfast was less frequent among elevated BMI-WHtR groups. Participants who consumed commercially baked goods or pastries for breakfast were less likely to be those in the ‘increased’ and ‘very high risk’ groups. Participants who took sweets and candies several times every day were more likely to be those in the ‘increased’ and ‘high risk’ groups. Participants who consumed cereals or grains products for breakfast and nuts regularly were less likely to be in the ‘very high risk’ group while participants who consumed raw or cooked vegetables more than once a day were more likely to be in this group.

## 4. Discussion

In the current study, 2.1% of NW, 40.4% of OW, and 90.6% of OB children and adolescents had abdominal obesity by WHtR. To analyze the magnitude of these data, these findings were compared with previous studies conducted in Spain [8,11], Turkey [10], and Bangladesh [9] that also analyzed the prevalence of abdominal obesity among BMI categories in children and/or adolescents. In a representative national sample of 1521 Spanish children and adolescents from 1998 to 2000, the respective prevalence of abdominal obesity was 7.5% of NW, 49.2% of OW, and 82.1% of OB children aged 6–11 years, and 1.8% of NW, 44.1% of OW, and 97.9% of OB adolescents aged 12–17 years [8]. The ENPE study (*Estudio Nutricional de la Población Española*) showed that the prevalences of abdominal obesity among NW, OW, and OB 9–18-year-old Spanish children and adolescents were 9.9%, 42.6%, and 84.0%, respectively [11]. Among 12–18-year-old Turkish girls, 2.6% of UW, 5.8% of NW, 37.3% of OW, and 77.3% of OB had abdominal obesity [10]. Among 9–17-year-old Bangladesh girls, 14% of NW, 46% of OW, and 54% of OB had abdominal obesity [9]. NW in combination with abdominal obesity was related to a higher risk of various metabolic risk indicators [30,31]. Nevertheless, the prevalence of abdominal obesity among NW, OW, and OB children and adolescents strongly depends on the definition of BMI categories and abdominal obesity [8].

Applying the WHO 2007 BMI criteria and defining abdominal obesity as WHtR ≥0.5, four BMI-WHtR risk categories were constructed. More than a third of the study population had high disease risk based on the combined BMI-WHtR classification. In a Norwegian adult population (aged 18–51 years) from the Telemark study, more than half of the participants represented combined BMI-WC categories with high disease risk [20]. In this Norwegian adult population study, there were more men than women in the BMI-WC ‘increased’ and ‘very high risk’ categories, while there were more women in the ‘high risk’ category [20]. The current study showed that there were more boys in the ‘very high risk’ group, which agrees with previous reports in which a higher prevalence of OW/OB and/or high WHtR were found among boys than girls [11,32]. This may possibly be due to stronger cultural and social pressure on girls to maintain an acceptable body image.

Participants ranked in the ‘very high risk’ category were 0.60 times lower among adolescents. Previous studies reported higher risk in a younger pediatric population [33,34]. In the current study, participants ranked in the ‘increased risk’ group were also 0.74 times lower among adolescents; however, the obesogenic behavior score was not positively associated in contrast with the ‘high’ and ‘very high risk’ categories. The likelihood of achieving the recommended physical activity level was only significantly reduced among the ‘high’ and ‘very high risk’ participants. Low physical activity was often associated with OB [35] and promoting physical activity remains important to ensure good health and well-being and to prevent chronic diseases [36,37]. The current participants in the ‘very high risk’ group did not significantly follow the recommended screen time on weekdays. The relationship between screen media exposure and obesity was widely studied, and the mechanisms linking screen media exposure and obesity described in the literature included increased eating while viewing; exposure to high-calorie low-nutrient food; beverage marketing that influences children’s preferences, purchase requests, and consumption habits; and low sleep duration [38].

No clear differences in adherence to the MedDiet were observed between the BMI-WHtR risk categories. Only participants who were ranked in the ‘high risk’ category showed lower moderate and optimal adherence to the MedDiet. Skipping breakfast was more frequent among participants with high BMI-WHtR disease risk, and hence they showed lower consumption of commercial baked goods or pastries for breakfast but also dairy products and cereal or grain products for breakfast. Several studies also pointed out those individuals who did not consume breakfast daily showed a high prevalence of overweight and obesity [39]. The mechanisms linking breakfast consumption to lower body weight are unclear. Skipping breakfast affects children’s appetite, but it does not necessarily imply the consumption of larger portion sizes at subsequent meals [40]. However, breakfast consumption may be associated with more regular eating habits and the selection of more healthful food choices [41]. Therefore, the current results highlight the need for interventions targeted at these elevated disease risk groups.

Participants who consumed sweets and candies several times every day were included in the ‘increased’ and ‘high risk’ groups. Previous studies showed that children and adolescents with general and/or abdominal obesity reported a low frequency of consumption of unhealthy foods (fast food, fries, cake, sugar-sweetened beverages, and sweets) [42]. Overweight and obese people could under-report their intake of unhealthy foods, but they also may pay more attention to foods that are energy dense [43]. Accordingly, participants who consumed raw or cooked vegetables more than once a day were 1.36 times more likely to be in the ‘very high risk’ group. Participants who consumed dairy products daily were lower in the ‘very high risk’ group and participants consuming nuts regularly were lower in the ‘high’ and ‘very high risk’ groups. Even though inconsistent findings for dairy products were observed [32,44,45], previous studies demonstrated inverse associations between dairy [32] and nuts [44,46] consumption and adiposity in children and adolescents. The mechanisms linking nut consumption to low body weight may include a high resting metabolic rate, enhanced satiety, low intake of other foods, and incomplete absorption of energy from nuts [44].

Individuals included in the ‘very high risk’ group were less likely to have mothers with high SDQS scores. Maternal high-quality diet was not associated with risk of obesity in offspring aged 9–14 years in mother–child pair studies conducted in the United States [47]. However, parental food intake was highly correlated with Chinese children and adolescent’s food intake, although this association between parental food intake and offspring’s overweight and obese was most significant in 7–12-year-old children compared to 13–18-year-old adolescents [48]. In New Zealand, higher parental diet quality was associated with lower consumption frequency of confectionary, chocolate, cakes, biscuits, and savory snacks in 9–11-year-old children [49]. Therefore, the current findings highlight the crucial role of maternal diet quality on child/adolescent BMI-WHtR disease risk.

Individuals in the ‘increased’ and ‘high risk’ categories had fewer mothers engaged in the physical activity recommendations, despite the risk of incident obesity being reported as being 0.79 times lower among offspring whose mothers engaged in at least 150 min/day of MVPA [47]. Significant associations between mothers’ lifestyle variables and child/adolescent BMI-WHtR disease risk were also observed for education level, BMI status, and smoking habit. Individuals in the ‘very high risk’ group had fewer mothers with high education levels. This relationship between the mother’s education level and overweight and obesity has been widely studied. The likelihood of having mothers with BMI <25 kg/m^2^ decreased with increasing BMI-WHtR disease risk level, as the BMI-WHtR categories showed. Obesity in parents is a well-known risk factor for weight problems in their offspring, and parental overweight or obesity is positively associated with abdominal obesity in children and adolescents [32,50,51]. Due to genetics and parental behavior, children with obese parents are at a greater risk of becoming obese themselves [52]. Individuals in the ‘very high risk’ group had fewer mothers that had never smoked or were former smokers. Childhood parental smoking exposure was previously associated with increased risk for life-course overweight or obesity and/or abdominal obesity [47,53].

### Strengths and Limitations

The strengths of this study were the relatively large and nationwide representative sample of children and adolescents, as well as the inclusion of relevant factors related to maternal history, which were not included in previous research on this topic. The current study has several limitations that should be recognized. Firstly, the PASOS study is cross-sectional research and, therefore it cannot establish causality for the significant associations studied. Secondly, except for children’s anthropometric data, other variables were obtained by means of questionnaires. Consequently, the findings show inherent limitations of self-reported data, such as memory bias and the influence of social desirability. Thirdly, BMI categories were estimated by applying the WHO 2007 criteria. Nevertheless, the International Obesity Task Force (IOTF) cut-off points yield different estimates of the prevalence of OW and OB, and it is unclear which provides the more accurate estimate. Fourthly, the two categories of obesity (WHtR_NO_ and WHtR_OB_) were collapsed to the ‘very high risk’ category due to small numbers (*n* = 51). Fifthly, not all the questions of the surveys were responded to by all the participants as well as all the mothers/female caregivers, which results in a reduced power due to the reduced sample size. However, the sample size is indicated in each used variable. The pattern of respondents who chose not to answer every question of this study was observed, and the pattern was random in relation to the participants’ BMI-WHtR categories except in relation to achieving the recommendation of <120 min/day of total screen time on weekdays and the BMI categories among mothers.

## 5. Conclusions

More than a third of the children and adolescents showed high disease risk categories. The current findings support the need to add WC into routine clinical practices, in addition to traditional measurements of weight and height. The identification of factors associated with combined BMI-WHtR categories associated with disease risks in children and adolescents drives a better understanding of their determinants and can guide prevention strategies for risk factors, as well as improve the overall health of this population. Mothers’ healthy lifestyle is associated with lower BMI-WHtR disease risk in their offspring. Therefore, the current findings support family intervention strategies to decrease childhood and adolescence disease risk associated with BMI and WHtR.

## Figures and Tables

**Table 1:** Descriptive characteristics of the study population relative to joint categories of body mass index (BMI) and waist-to-height ratio (WHtR).

Variables			Joint Categories of BMI and WHtR				
	*n*	Total	Low Risk BMI_NW_–WHtR_NO_	Increased Risk BMI_OW_–WHtR_NO_	High Risk BMI_NW-OW_–WHtR_OB_	Very High Risk BMI_OB_–WHtR_NO-OB_	*p*-Value
*n*	3772	3772	2402	464	366	540	
Age (y-o) ^†^	3772	12.5 ± 2.4	12.7 ± 2.4 ^a,c^	12.2 ± 2.3 ^a,d^	12.8 ± 2.4 ^d,f^	12.0 ± 2.3 ^c,f^	<0.001
<12 ^‡^	1707	45.3	42.4 ^a,c^	50.0 ^a^	42.6 ^f^	55.6 ^c,f^	<0.001
≥12 ^‡^	2065	54.7	57.6	50.0	57.4	44.4	
Sex							
Male ^‡^	1836	48.7	46.3 ^c^	49.6 ^e^	49.5 ^f^	58.0 ^c,e,f^	<0.001
Female ^‡^	1936	51.3	53.7 ^c^	50.4 ^e^	50.5 ^f^	42.0 ^c,e,f^	
Number of people at home ^†^	3238	3.5 ± 1.6	3.5 ± 1.5	3.5 ± 1.1	3.6 ± 2.9	3.5 ± 1.3	0.418
2 persons ^‡^	543	16.8	17.3	14.8	16.5	16.1	0.219
3–4 persons ^‡^	2272	70.2	70.7	70.6	69.7	67.6	
≥5 persons ^‡^	423	13.1	12.0	14.6	13.8	16.3	
MVPA (min/day) ^†^	3605	125.4 ± 78.3	126.8 ± 78.7 ^b^	134.2 ± 77.6 ^d^	112.6 ± 76.3 ^b,d^	120.9 ± 77.0	0.001
MVPA ≥ 60 min/day ^‡^	2858	79.3	79.8 ^b^	82.7 ^d^	72.8 ^b,d^	78.8	0.005
Total screen time weekdays (min/day) ^†^	3565	179.6 ± 147.1	177.2 ± 145.8 ^c^	161.1 ± 142.8 ^d,e^	189.9 ± 149.5 ^d^	198.6 ± 152.5 ^c,e^	<0.001
Screen time weekdays < 120 min/day ^‡^	1611	45.2	45.5 ^a^	53.4 ^a,d,e^	41.7 ^d^	39.4 ^e^	<0.001
Total screen time weekends (min/day) ^†^	3565	283.1 ± 172.9	281.2 ± 171.6 ^c^	263.5 ± 170.0 ^e^	288.1 ± 170.8	304.5 ± 180.9 ^c,e^	0.003
Screen time weekends < 120 min/day ^‡^	734	20.6	20.5	24.5	18.5	19.1	0.120
KIDMED index ^†^	3576	6.8 ± 2.5	6.8 ± 2.5 ^b^	7.1 ± 2.5 ^d,e^	6.4 ± 2.5 ^b,d^	6.6 ± 2.4 ^e^	0.001
Low ^‡^	365	10.2	9.6	9.4 ^d^	14.0 ^d^	11.0	0.020
Moderate ^‡^	1771	49.5	50.1	44.5	49.3	51.4	
Optimal ^‡^	1440	40.3	40.3	46.1	36.7	37.6	
Obesogenic behavior score (0–3) ^†^	3557	1.0 ± 0.8	1.0 ± 0.8 ^b^	0.9 ± 0.8 ^d,e^	1.2 ± 0.8 ^b,d^	1.1 ± 0.8 ^e^	<0.001
0 ^‡^	933	26.2	26.5 ^a^	33.9 ^a,d,e^	21.6 ^d^	21.7 ^e^	<0.001
1 ^‡^	1760	49.5	50.3 ^a^	42.7 ^a,e^	48.7	52.3 ^e^	
2 ^‡^	748	21.0	20.2	21.1	23.5	23.1	
3 ^‡^	116	3.3	3.1 ^b^	2.3 ^b^	6.2 ^b,d^	3.0	

Data are shown as ^†^ means ± standard deviations or ^‡^ percentages. Abbreviations: y-o, years old; MVPA, moderate and vigorous physical activity; min, minutes; BMI, body mass index; WHtR, waist-to-height ratio; BMI_NW_, normal-weight; WHtR_NO_, abdominal non-obesity; BMI_OW_, overweight; WHtR_OB_, abdominal obesity; BMI_OB_, obesity. Differences in means were tested by the ANOVA test and differences in percentages by Chi-squared test. Different letters in rows shows statistically significant differences between categories of BMI and WHtR by the Bonferroni post-hoc test: ^a^: low risk vs. increased risk; ^b^: low risk vs. high risk; ^c^: low risk vs. very high risk; ^d^: increased risk vs. high risk; ^e^: increased risk vs. very high risk; ^f^: high risk vs. very high risk.

**Table 2:** Descriptive maternal characteristics of the study population relative to joint categories of body mass index (BMI) and waist-to-height ratio (WHtR).

Variables			Joint Categories of BMI and WHtR				
	*N*	Total	Low Risk BMI_NW_–WHtR_NO_	Increased Risk BMI_OW_–WHtR_NO_	High Risk BMI_NW-OW_–WHtR_OB_	Very High Risk BMI_OB_–WHtR_NO-OB_	*p*-Value
Maximum education level ^‡^							
University	1047	31.3	33.1 ^c^	33.9 ^e^	28.9	22.3 ^c,e^	<0.001
Secondary	1811	54.1	53.2	53.4	56.3	57.2	
Primary/Illiterate	490	14.6	13.7 ^c^	12.7 ^e^	14.8	20.6 ^c,e^	
SDQS score ^†,‡^	958	39.0 ± 3.1	39.2 ± 3.1 ^c^	39.7 ± 3.2 ^e^	38.7 ± 3.1	38.0 ± 2.9 ^c,e^	<0.001
T1: 0.0–37.9	305	31.8	30.2 ^c^	23.6 ^e^	32.6	47.5 ^c,e^	0.003
T2: 38.0–39.9	232	24.2	23.8	27.3	29.1	20.0	
T3: 40.0–49.0	421	43.9	46.0 ^c^	49.1	38.4	32.5 ^c^	
BMI categories (kg/m^2^) ^‡^							
Normal weight	1794	57.7	65.1 ^a,b,c^	53.5 ^a,e^	46.5 ^b,f^	34.9 ^c,e,f^	<0.001
Overweight	905	29.1	26.0 ^b,c^	31.2	36.4 ^b^	36.5 ^c^	
Obesity	411	13.2	9.0 ^a,b,c^	15.3 ^a,e^	17.1 ^b,f^	28.6 ^c,e,f^	
Smoking habit ^‡^							
Smoker	797	23.8	21.8 ^c^	22.8 ^e^	26.3	31.8 ^c,e^	<0.001
Former smoker	832	24.8	25.4	27.8	23.0	20.6	
Never smoked	1725	51.4	52.8	49.4	50.7	47.5	
Screen time weekend (min/day) ^†^	3119	179.4 ± 107.8	176.9 ± 104.0	177.7 ± 107.7	184.0 ± 113.2	189.4 ± 120.1	0.138
Screen time weekends < 120 min/day ^‡^	1383	44.3	45.6	46.3	40.9	39.3	0.049
Screen time weekdays (min/day) ^†^	3156	123.7 ± 96.7	122.3 ± 94.2	121.8 ± 100.3	123.9 ± 95.3	131.9 ± 105.5	0.307
Screen time weekdays < 120 min/day ^‡^	2342	74.2	74.7	75.6	75.0	70.3	0.243
Total PA(METs × min/day) ^†^	2162	444.0 ± 623.8	452.2 ± 621.9	374.1 ± 567.6	429.5 ± 662.5	476.5 ± 646.8	0.212
PA ≥ 300 METs × min/day ^‡^	974	45.1	47.2 ^a,b,c^	39.7 ^a,e^	39.4 ^b,f^	44.3 ^c,e,f^	0.037

Data are shown as ^†^ means ± standard deviations or ^‡^ percentages. Abbreviations: SDQS, short Diet Quality Screener; T1, tertile 1; T2, tertile 2; T3, tertile 3; kg, kilograms; m, meters; min, minutes; PA, physical activity; MET, metabolic equivalents; BMI, body mass index; WHtR, waist-to-height ratio; BMI_NW_, normal-weight; WHtR_NO_, abdominal non-obesity; BMI_OW_, overweight; WHtR_OB_, abdominal obesity; BMI_OB_, obesity. Differences in means were tested by the ANOVA and differences in percentages by Chi-squared test. Different letters in rows shows statistically significant differences between categories of BMI and WHtR by the Bonferroni post-hoc test: ^a^: low risk vs increased risk; ^b^: low risk vs high risk; ^c^: low risk vs very high risk; ^e^: increased risk vs very high risk; ^f^: high risk vs very high risk.

**Table 3:** Odds ratios (and 95% confidence intervals) from logistic regression showing the correlation of characteristics of child/adolescent and their mothers/female caregivers with children’s categories of BMI and WHtR.

	Joint Categories of BMI and WHtR		
	Increased Risk BMI_OW_–WHtR_NO_	High Risk BMI_NW-OW_-WHtR_OB_	Very High Risk BMI_OB_–WHtR_NO-OB_
Child/adolescent characteristics			
Age			
<12 years	1.00 (ref.)	1.00 (ref.)	1.00 (ref.)
≥12 years	0.74 (0.61–0.90) **	1.00 (0.80–1.24)	0.60 (0.49–0.72) ***
Sex			
Male	1.00 (ref.)	1.00 (ref.)	1.00 (ref.)
Female	0.88 (0.72–1.08)	0.88 (0.71–1.10)	0.63 (0.52–0.76) ***
MVPA ≥ 60 min/day			
No	1.00 (ref.)	1.00 (ref.)	1.00 (ref.)
Yes	1.07 (0.81–1.41)	0.65 (0.50–0.85) **	0.73 (0.57–0.93) *
Screen time weekdays < 120 min/day			
No	1.00 (ref.)	1.00 (ref.)	1.00 (ref.)
Yes	1.23 (0.99–1.54)	0.86 (0.67–1.10)	0.61 (0.49–0.76) ***
KIDMED index			
Low	1.00 (ref.)	1.00 (ref.)	1.00 (ref.)
Moderate	0.90 (0.62–1.30)	0.67 (0.48–0.95) *	0.89 (0.64–1.23)
Optimal	1.11 (0.77–1.61)	0.62 (0.43–0.89) **	0.76 (0.55–1.07)
Obesogenic behavior score (0–3)			
0	1.00 (ref.)	1.00 (ref.)	1.00 (ref.)
1	0.73 (0.57–0.94) *	1.23 (0.91–1.66)	1.56 (1.21–2.02) **
2	1.00 (0.73–1.38)	1.57 (1.09–2.25) *	2.19 (1.60–3.01) ***
3	0.80 (0.39–1.63)	2.86 (1.61–5.10) ***	2.52 (1.34–4.72) **
Mother/female legal guardian			
Maximum education level			
Primary/Illiterate	1.00 (ref.)	1.00 (ref.)	1.00 (ref.)
Secondary	1.06 (0.77–1.48)	0.98 (0.70–1.38)	0.71 (0.54–0.93) *
University	1.06 (0.75–1.49)	0.81 (0.56–1.17)	0.43 (0.31–0.58) ***
SDQS score			
T1: 0.0–37.9	1.00 (ref.)	1.00 (ref.)	1.00 (ref.)
T2: 38.0–39.9	1.44 (0.82–2.55)	1.14 (0.63–2.03)	0.52 (0.31–0.88) *
T3: 40.0–49.0	1.38 (0.83–2.29)	0.79 (0.46–1.34)	0.44 (0.28–0.70) ***
BMI categories (kg/m^2^)			
Obesity	1.00 (ref.)	1.00 (ref.)	1.00 (ref.)
Overweight	0.68 (0.48–0.97) *	0.73 (0.50–1.05)	0.41 (0.30–0.55) ***
Normal weight	0.46 (0.33–0.64) ***	0.37 (0.26–0.53) ***	0.16 (0.12–0.21) ***
Smoking habit			
Smoker	1.00 (ref.)	1.00 (ref.)	1.00 (ref.)
Former smoker	1.07 (0.79–1.44)	0.76 (0.54–1.05)	0.57 (0.43–0.76) ***
Never smoked	0.92 (0.70–1.20)	0.81 (0.61–1.06)	0.64 (0.51–0.81) ***
Screen time weekends < 120 min/day			
No	1.00 (ref.)	1.00 (ref.)	1.00 (ref.)
Yes	0.99 (0.80–1.24)	0.82 (0.64–1.05)	0.74 (0.60–0.92) **
PA ≥ 300 METs × min/day			
No	1.00 (ref.)	1.00 (ref.)	1.00 (ref.)
Yes	0.75 (0.57–0.98) *	0.73 (0.54–0.97) *	0.90 (0.70–1.15)

Abbreviations: min, minutes; kg, kilograms; m, meters; MVPA, moderate and vigorous physical activity; BMI, body mass index; WHtR, waist-to-height ratio; BMI_NW_, normal weight; WHtR_NO_, abdominal non-obesity; BMI_OW_, overweight; WHtR_OB_, abdominal obesity; BMI_OB_, obesity. Odds ratio (OR) with 95% confidence intervals (CI) were calculated by binary logistic regression analysis. The analysis was adjusted by sex and age unless the variable was the one of interest. Reference group = ‘low risk’ (NW BMI-WHtR_NO_); OR presented for ‘low risk’ vs. ‘increased’, ‘high’ or ‘very high risk’. *p*-value: * *p* < 0.05; ** *p* < 0.01; *** *p* < 0.001.

**Table 4:** Odds ratios (and 95% confidence intervals) from logistic regression showing the correlation of children’s “yes” answers in the KIDMED questionnaire and children’s categories of BMI and WHtR.

KIDMED Questionnaire		Joint Categories of BMI and WHtR		
	Low Risk BMI_NW_–WHtR_NO_	Increased Risk BMI_OW_–WHtR_NO_	High Risk BMI_NW-OW_–WHtR_OB_	Very High Risk BMI_OB_–WHtR_NO-OB_
	*n* = 2273	*n* = 438	*n* = 357	*n* = 508
Q1. Skips breakfast (−)	1.00 (ref.)	1.44 (1.06–1.95) *	1.46 (1.06–2.01) *	1.67 (1.26–2.23) ***
Q2. Has dairy product for breakfast (+)	1.00 (ref.)	0.67 (0.52–0.86) **	0.64 (0.49–0.84) **	0.66 (0.52–0.84) **
Q3. Has cereal or grains product for breakfast (+)	1.00 (ref.)	0.90 (0.73–1.12)	0.79 (0.63–1.00)*	0.75 (0.62–0.92) **
Q4. Pastries/commercially baked goods for breakfast (−)	1.00 (ref.)	0.67 (0.53–0.85) **	0.82 (0.64–1.04)	0.68 (0.55–0.84) **
Q5. Takes a fruit or fruit juice daily (+)	1.00 (ref.)	1.20 (0.95–1.53)	0.83 (0.65–1.06)	1.02 (0.82–1.27)
Q6. Has a second serving of fruit daily (+)	1.00 (ref.)	1.03 (0.84–1.27)	0.88 (0.70–1.10)	0.98 (0.80–1.19)
Q7. Two yogurts and/or 40 g cheese daily (+)	1.00 (ref.)	0.98 (0.77–1.25)	0.83 (0.64–1.07)	0.79 (0.63–0.98) *
Q8. Has fresh or cooked vegetables daily (+)	1.00 (ref.)	1.24 (1.00–1.55)	0.86 (0.69–1.08)	0.99 (0.81–1.22)
Q9. Has fresh or cooked vegetables more than 1/day (+)	1.00 (ref.)	1.16 (0.93–1.44)	1.09 (0.86–1.38)	1.36 (1.11–1.67) **
Q10. Regular fish consumption (at least 2–3/week) (+)	1.00 (ref.)	1.10 (0.89–1.37)	0.82 (0.65–1.03)	0.87 (0.71–1.06)
Q11. Goes >1/week fast food restaurant (−)	1.00 (ref.)	0.77 (0.60–1.00)	0.89 (0.68–1.17)	1.11 (0.89–1.39)
Q12. Regular nut consumption (≥2–3/week) (+)	1.00 (ref.)	0.97 (0.79–1.19)	0.77 (0.62–0.97) *	0.69 (0.57–0.84) ***
Q13. Likes pulses and eats more than 1/week (+)	1.00 (ref.)	1.26 (1.00–1.59)	0.83 (0.65–1.05)	1.00 (0.81–1.24)
Q14. Takes sweets and candies several times every day (−)	1.00 (ref.)	0.58 (0.44–0.77) ***	0.71 (0.54–0.95) *	0.87 (0.69–1.10)
Q15. Consumes rice or pasta almost daily (≥5/week) (+)	1.00 (ref.)	0.97 (0.79–1.19)	0.94 (0.75–1.18)	0.95 (0.78–1.16)
Q16. Uses of olive oil at home (+)	1.00 (ref.)	0.84 (0.59–1.19)	0.74 (0.50–1.08)	0.77 (0.56–1.07)

Abbreviations: Q, question; BMI, body mass index; WHtR, waist-to-height ratio; BMI_NW_, normal weight; WHtR_NO_, abdominal non-obesity NW, normal-weight; BMI_OW_, overweight; WHtR_OB_, abdominal obesity; BMI_OB_, obesity. (+): positive value on KIDMED score; (−): negative value on KIDMED score. Odds ratio (OR) with 95% confidence intervals (CI) were calculated by binary logistic regression analysis. The analysis was adjusted by sex and age. Reference group = “no”; OR presented for “no” vs. “yes”. In the present analysis, 3576 participants were included (and 196 were excluded since they have missing data). *p*-value: * *p* < 0.05; ** *p* < 0.01; *** *p* < 0.001.

## Data Availability

There are restrictions on the availability of data for this trial, due to the signed consent agreements around data sharing, which only allow access to external re-searchers for studies following the project purposes. Requestors wishing to access the trial data used in this study can make a request to pep.tur@uib.es.
